# Regeneration in the phylogenetic empire: an interview with Peter Currie

**DOI:** 10.1242/dmm.049404

**Published:** 2021-12-23

**Authors:** Peter Currie


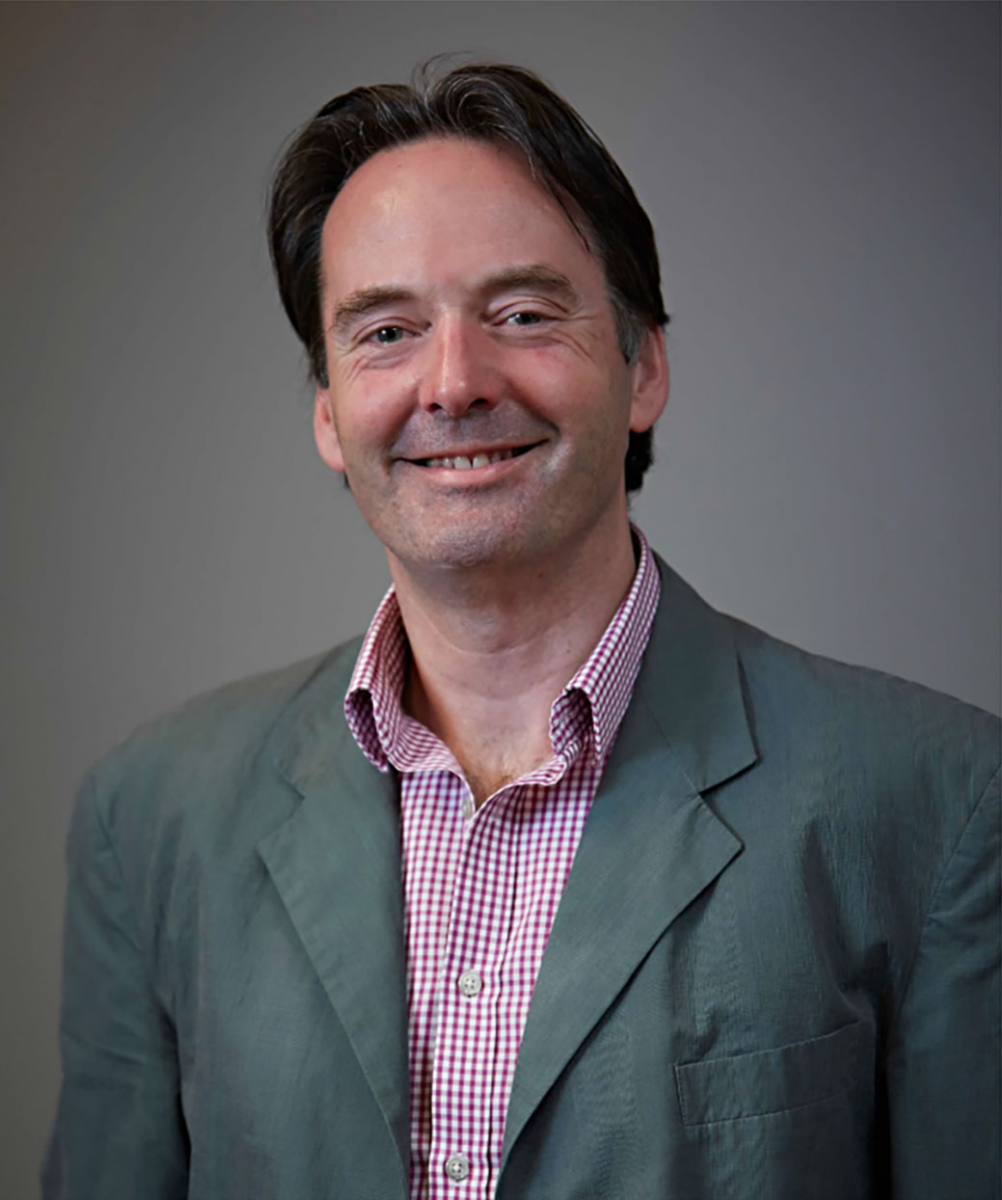


Professor Peter Currie is a world-leading developmental, evolutionary and stem cell biologist. His work examines the development and regeneration of skeletal muscles through stem cell activity and applies this to human muscle disease. He pioneered the use of zebrafish as a model for muscle disease and regeneration, and he was also integral in the revival of shark embryos as a tool to study development at an evolutionary level.

Peter began his career studying *Drosophila* genetics during his PhD in Dr David Sullivan's laboratory at Syracuse University, New York. He then shifted from air to aquatics as he explored zebrafish development during his postdoctoral training in Dr Philip Ingham's laboratory at the Imperial Cancer Research Fund (now Cancer Research UK) in London. He launched his independent laboratory at the UK Medical Research Council Human Genetics Unit in Edinburgh and then moved to the Victor Chang Cardiac Research Institute in Sydney, Australia. In 2016, he was appointed Director of Research of the Australian Regenerative Medicine Institute at Monash University in Melbourne. He is also Head, EMBL Australia Melbourne Node and a senior principal research fellow with the National Health and Medical Research Council in Australia. In this interview, Peter discusses his development of novel laboratory model systems throughout his career, the potential clinical applications of his discoveries, and his vision for the future of developmental, regenerative and evolutionary biology.

**What makes zebrafish a good model for studying human muscle disease and regeneration?**

My interest in zebrafish stems back to my post-PhD days when zebrafish were just emerging as a model. I wanted to answer vertebrate-specific problems that were relevant to human disease, but I was very much weighted to a genetic approach, because I had trained using *Drosophila* and *Caenorhabditis elegans*. I really wanted to find a vertebrate model system in which I could use invertebrate-style genetic approaches. I went to several of the labs in Boston that were studying zebrafish at the time, such as the one of Nancy Hopkins, who generously spent time showing me around. I was amazed at the optical clarity of the zebrafish embryo and the scale of the genetic approaches being deployed. Then, when I began my postdoctoral training, I realised the power of doing *in vivo* cell biology in a system that had pliable genetics. I became very fascinated with imaging technologies, and their ability to answer not only developmental questions but also disease-impacting questions in living tissues within an animal. The combination of genetics and *in vivo* biology really sustained my interest in the system for over 30 years now.

“The great strength of the zebrafish system is that not only do we have a system in which you can see biological processes happening in real time, and you can genetically dissect these processes, but it also has some unique features of its biology that are really impactful.”

**You have incredible microscopy videos of zebrafish stem cells migrating to an injury, even a severed spinal cord, and repairing the wound. How could we potentiate this kind of activity in human stem cells?**

Yes, so that is a really amazing feature of regenerative vertebrates, such as zebrafish and Axolotls [a type of aquatic salamander]. The endless questions that are asked of regenerative biologists who work in these systems are “how can they do it?” and “wouldn't it be amazing if we could regenerate tissue like them?”. The great strength of the zebrafish system is that not only do we have a system in which you can see biological processes happening in real time, and you can genetically dissect these processes, but it also has some unique features of its biology that are really impactful. I always liken this type of conversation to trying to build a car from scratch with all the components laid out without a manual, which would be a trial-and-error process. Whereas if you have a model system or a manual that shows you how it can be done, then that accelerates your ability to achieve your goal. So that's what I think zebrafish and Axolotl are: they are a roadmap of how to potentiate regeneration in non-regenerating tissues. Once you understand how that's achieved, then you can apply it in settings where it's not currently being achieved.

More recently, I've been fascinated by the immune system as the gatekeeper of regeneration. I think the immune system is a very powerful paradigm for human disease because we know so much about it. There are so many ways to manipulate the immune system with drugs, making it a very amenable system to unlocking regenerative potential of human cells.

**Following on, how does human skin and liver have much greater regenerative potential than other tissues?**

Conceptually, why some tissues can regenerate, and even why some organisms can regenerate better than others, is a real conundrum. There is no unifying theory that explains differences in regenerative capacity across the phylogeny or within an individual organism. Even within some animals, the way that regeneration occurs is completely different depending on the tissue. For instance, skeletal muscle in zebrafish has a well-defined stem cell system called the satellite cell, which has a direct analogous cell type in mammals, whereas regeneration of the heart in zebrafish, which is not a capacity mammals have, occurs by dedifferentiation of the tissue. Interestingly, liver is one of the tissues in mammals that is able to dedifferentiate as well. So, differences in regenerative capacity in certain tissues are not always because they don't harbour a tissue-resident stem cell, but because different tissues regenerate using different mechanisms. More recently, we began to realise that the simple stem cell systems that have been portrayed in many mammalian tissues are actually not as straightforward or linear in their self-renewal characteristics as had previously been suspected.

So, the big answer to that question is we haven't got the faintest idea. It is really one for the ages. My old boss Nadia Rosenthal used to say that humans' lack of regenerative capacity was maybe because we were just ‘losers’. I do like that from a phylogenetic perspective, and evolution does hold some of the keys to this question. I think this question can only be tackled by going back through the evolution of tissues and their regenerative capacities to understand why these (in)capabilities have arisen. Each mechanism that has evolved is for a particular metabolic or tissue function that's selected for within a lineage and maintained or lost depending on evolutionary pressure. I think you could say that about any tissue function, but I honestly think that is the most logical explanation. But *why* they've evolved and been lost, when clearly most tissues and organisms retaining regenerative capacity would seemingly to be evolutionary advantageous, is difficult to know. One theory that's been advanced is that the immune system has evolved to be more complex in mammals than in some fish species, and there is a trade-off between immune complexity and flexibility to regenerate tissues. I'm not sure that theory has a lot of evidence, but it is present in the literature. However, we have the ability to answer some of those immune centric questions in our lab systems.

**Which discovery from your career has the potential to make the biggest impact for people with muscular disease?**

That's an interesting concept. I suppose most scientists, when they reach a certain point in their career, like I have, would love to see their discoveries make real-world impact. Simply discovering and gathering the knowledge is not sufficient, and more intellectual curiosity arises from how a discovery can be implemented. I think our fundamental discovery that zebrafish can model human muscle disease accurately will probably have the longest impact on translation, because it's led to the uptake of that model for disease modelling and drug discovery purposes. Although we won't be able to draw a direct line for citations or drug discovery, I think, conceptually, that discovery set the field for using zebrafish in muscle disease modelling.

The most obvious discovery, however, is the role of the macrophage-secreted cytokine Nampt in muscle regeneration (Ratnayake et al., 2021), as it has direct commercial potential, and there's been a lot of interest in this from industry. There's also a lot of interest in macrophages as a transient stem cell niche, which can impact a variety of different stem cell populations and tissues. So those two angles will probably have the quickest route to the clinic.

Other discoveries were made in the laminin alpha 2-deficient zebrafish model of congenital muscular dystrophy type 1A (MDC1A) (Hall et al., 2019; Wood et al., 2019). MDC1A is a very impactful dystrophy, as it often causes severe illness in the newborn. In the zebrafish, we created a concept of muscle fibre stabilisation and re-functionalisation that could have therapeutic potential. There was also a stem cell deficit in that model, and supplementation with laminin was shown to be a therapeutic approach that was also effective in mouse models.

“When I picked up these shark embryos, took them back to the lab and cut one open, there was the most exquisite embryo you've ever seen in your entire life. It was absolutely drop-dead gorgeous.”

**Why did you decide to start exploring the evolution of regenerative biology in shark embryos?**

My fascination in evolution stems from a broad interest in studying biology from a diversity perspective. I think we know an awful lot about very few animals, and I think we're a lot poorer for it. The reason that I'm interested in sharks and basal Gnathostomes [jawed, bony fish] is because they're a really critical node in the evolution of complex tissues. You can chart many developmental mechanisms back to Gnathostomes [including sharks], such as the formation of the head.

As I was at Lincoln's Inn Field [the site of the Cancer Research UK labs], during my postdoc, we were actually directly across from the science component of the British Library. The literature there on muscle formation in sharks was all in scientific German from two centuries ago. Once these references had been translated by my German friends, they said that sharks had a completely different developmental mechanism than what had recently been published in mouse. At the turn of the last century, there was quite a lot of embryology research in sharks because people knew the phylogenetic position of sharks was so important. But, because of the modern era of genetics, those types of systems have been completely discarded from developmental studies. So, I just asked a simple question: were the pictures in these dusty old books from the British Library in any way accurate, or did they reflect an inability to do hardcore developmental biology? So, when I moved to Edinburgh, I found out that there's a really good Marine Station nearby, in Oban, and I thought it must be possible to get shark embryos. I asked whether they'd be able to collect the uterus of a relatively small shark species. Then I got an email from the guy on the marine survey vessel saying, “Your little collection tubes are too small”. I thought it was a bit strange, but it turned out they'd collected the uterus from another species, which was almost five feet long. Then, obviously, I had to do more research finding which species to work with and I settled on *Scyliorhinus canicula*, which is the dogfish. When I picked up these shark embryos, took them back to the lab and cut one open, there was the most exquisite embryo you've ever seen in your entire life. It was absolutely drop-dead gorgeous. Then there was no competition – I was working with these things. I thought that this was the first time a shark embryo had been opened in a research lab for probably 80 years, but little did I know that Cheryll Tickle was also working on sharks at the University of Dundee. We just didn't realise at the time that we were both working on them. And so, that was the birth of shark work in my lab, and that was the first ever paper I published from my own lab.

Nick Hastie, the head of the human genetics unit in Edinburgh, did look at me rather strangely when I brought the shark embryos into the lab, but I did convince him that it was relevant to human disease. Since then, we've been trying to use them to understand the origins of some of the questions that we talked about at the start of the interview. We're particularly interested in the fin-to-limb transition and how complexity has arisen, and shark embryos have been really very important for understanding aspects of that. In Australia, we have now established some of the world's best husbandry practices for shark species, and we have a colony of epaulette sharks. This colony has 30 adults and we're producing hundreds of eggs every year. This is a really fun and rewarding part of the lab, and we think we've created a really amenable shark model system now.

**If you had unlimited time and resources, what grand experiment would you do?**

I would like to build my phylogenetic empire. We currently have five species we're working on, but I would build the world's biggest aquarium and I would start sampling diversity and regenerative capacity and development across key species in the phylogeny. I think there's a real opportunity to apply the amazing techniques in transcriptomics, such as spatial transcriptomics and single-cell sequencing, in a phylogenetic context. My big dream would be to try to tackle some of the problems in regenerative and developmental research, across the phylogeny, with the genetic depth and sophistication that we have in different model systems, and start developing a broader understanding of the way evolution works to sculpt regeneration and tissue formation.

“My science has had a very positive uplift from becoming director of the [Australian Regenerative Medicine] Institute, because the younger scientists come in and keep my science in the 21st century.”

**How important is it to create an international network with fellow researchers?**

Well, it's absolutely critical. When conceptualising your ideas in the context of the field you can't put your head in the sand. In isolation, you can certainly come up with ideas and concepts that are unique to your own understanding and intellectual thread, but it's absolutely critical that you test those, you get feedback and you analyse them critically. I've desperately missed the scientific meetings that I routinely went to before the COVID-19 pandemic. They are really important to me, because I'm very embedded in the international muscle community; they are my friends and colleagues. I normally show unpublished data in these meetings, because it's a very trusting and safe community. For instance, I talked about the project investigating NAMPT (Ratnayake et al., 2021) 2 years before publication, and I got a lot of positive feedback and a lot of ideas about it. That really lifted the paper as it got braver and tackled some mammalian biology as well. All of those things enhance your science. My lab, like most labs, strives to be is interdisciplinary, but you can't conquer all disciplines simultaneously, and the quickest way to do that is collaboratively. If you look at my papers, the ones that are published in good journals nearly always carry two or three really key collaborators on them.

One of the best things about being the director of an institute is when you're recruiting people, you really have to understand what they do, and you have to understand technically what they bring to the institute. Once you go through that process, you realise how that can inform your own science. My science has had a very positive uplift from becoming director of the [Australian Regenerative Medicine] Institute, because the younger scientists come in and keep my science in the 21st century. A lot of them are also collaborators on my papers, and new group leaders that come to the institute have perhaps been the most influential part of my collaborative network. Being part of the creation of an institute that's only 10 or 12 years old and having everybody coming here with relatively new, shiny ideas and techniques has really had a positive influence on my personal science.

Aside from my collaborations, when we recruited a bunch of younger group leaders together at the institute, they immediately realised that if they collaborated together, they would each get to their individual goals much quicker. They each had a skill set that they've contributed and there's been some amazing work come out of the Institute. The Institute has really benefited from this collaborative environment.

**You're obviously very dedicated to your work, but is there anything you do outside of work that revitalises your energy for research?**

I'm a very keen student of history, particularly classical and Roman history. I bore my children with endless stories of what the Romans would have done in particular circumstances, and I love going to classical monuments. The other thing I do is draw, which has sustained me over the COVID-19 lockdowns. The problem with lockdowns is that you can spend all day working because there's no graduation between your working and personal lives. So, I make sure that I've got other projects on the go. I'm also a very keen snorkeler and diver, and I spend every moment, when the weather is well enough, with my head down and my bum up in the water, looking at fish. So, they are three things I do to try to maintain my sanity.

## References

[DMM049404C1] Hall, T. E., Wood, A. J., Ehrlich, O., Li, M., Sonntag, C. S., Cole, N. J., Huttner, I. G., Sztal, T. E. and Currie, P. D. (2019). Cellular rescue in a zebrafish model of congenital muscular dystrophy type 1A. *NPJ Regen. Med.* 4, 21. 10.1038/s41536-019-0084-5 31754462 PMC6858319

[DMM049404C2] Ratnayake, D., Nguyen, P. D., Rossello, F. J., Wimmer, V. C., Tan, J. L., Galvis, L. A., Julier, Z., Wood, A. J., Boudier, T., Isiaku, A. I. et al. (2021). Macrophages provide a transient muscle stem cell niche via NAMPT secretion. *Nature* 591, 281-287. 10.1038/s41586-021-03199-7 33568815

[DMM049404C3] Wood, A. J., Cohen, N., Joshi, V., Li, M., Costin, A., Hersey, L., McKaige, E. A., Manneken, J. D., Sonntag, C., Miles, L. et al. (2019). RGD inhibition of itgb1 ameliorates laminin-α2-deficient zebrafish fibre pathology. *Hum. Mol. Genet.* 28, 1403-1413. 10.1093/hmg/ddy426 30566586

